# ﻿Biodiversity of Italian freshwaters: an updated checklist of mayfly species (Ephemeroptera) as a starting point for the next taxonomic (r)evolution

**DOI:** 10.3897/zookeys.1239.147826

**Published:** 2025-05-28

**Authors:** Andrea Buffagni, Carlo Belfiore

**Affiliations:** 1 CNR-IRSA, National Research Council, Water Research Institute, Via del Mulino 19, 20871 Brugherio (MB), Italy National Research Council, Water Research Institute Brugherio Italy; 2 NBFC, National Biodiversity Future Center, Viale delle Scienze, 90128 Palermo, Italy National Biodiversity Future Center Palermo Italy; 3 DEB, Department of Biological and Ecological Sciences, Tuscia University, Viterbo, Italy Tuscia University Viterbo Italy

**Keywords:** Endemic species, identification uncertainty, Italy, Nature Restoration Law, taxonomy

## Abstract

The study of biodiversity and ecosystems must be based on detailed knowledge of animal and plant organisms. European strategies for the protection of biodiversity and the restoration of natural environments advocate in-depth studies where knowledge is most lacking or fragmentary, in order to adequately assess relevant changes. Italian Ephemeroptera have not been the subject of specific taxonomic studies for approximately 30 years. This paper presents a list of the species currently thought to occur in Italy, which amounts, on the basis of morphology, to 106 species. Of these, approximately one-fifth are endemic species, demonstrating the importance of the Italian territory for European and Mediterranean biodiversity. The main critical aspects are discussed with reference to both the recent history of the study of Italian Ephemeroptera and the prospects for future development. The general picture shows that i) many species are insufficiently studied and known; ii) there are probably several new species to be described; iii) several endemic species described in the last decades or centuries require confirmation of validity and/or presence. Specific studies on mayflies will need to focus on these aspects in order to increase our knowledge of natural systems and enable their appropriate protection and restoration.

## ﻿Introduction

Where species-level organism identifications are available, evidence indicates that global changes are driving widespread extinctions at unprecedented rates across all groups of organisms (Butchart et al. 2010; Román-Palacios and Wiens 2020). Drawing on such findings, the European Commission has established the EU Biodiversity Strategy (EC 2021), which seeks to protect nature and reverse ecosystem degradation. The Mediterranean region, with Italy at its core, stands out for its rich biodiversity. This is largely attributed to its unique combination of climatic, biogeographical, and land fragmentation conditions, which support a high number of endemic species compared to other European and non-European regions (Myers et al. 2000; Perret et al. 2023). Italy is home to several key biodiversity hotspots, including the Sardinian-Corsican complex, the mountainous Apennine regions, and Sicily (e.g., Bisconti et al. 2016; Martino et al. 2022). Freshwater ecosystems, particularly rivers, play an essential role in sustaining biodiversity (Bonada et al. 2007). Among the most abundant organisms in riverine environments are insects of the order Ephemeroptera, which represent a significant portion of both biomass (Sartori and Brittain 2015) and species diversity (Jacobus et al. 2019). Despite this, the study of Ephemeroptera taxonomy in Italy has largely remained at an exploratory stage, with a few exceptions focused on specific genera and geographic areas. Taxonomic knowledge is a cornerstone for understanding biodiversity, as detailed knowledge—at the species level—is essential for studying organisms and communities in depth (Lenat and Resh 2001). Italian Ephemeroptera have been the subject of intensive taxonomic research during only two relatively brief periods. The first occurred in the 1950s (e.g., Grandi 1951, 1959; Biancheri 1959), and the second during the 1980s and 1990s (e.g., Belfiore 1981, 1987, 1990, 1995). To date, a total of 18 Ephemeroptera species have been described from specimens collected in Italy by Italian authors. Together with another three species from Italy described by foreign authors, this represents ~ 1/5 of all Ephemeroptera species currently known to occur in Italy. Reflecting Italy’s importance for European and Mediterranean biodiversity, almost 90% of the species described from Italy are considered endemic (Bauernfeind and Soldán 2012; Wagner et al. 2017; Yanai et al. 2022). This makes Italy a perfect candidate for priority funding of curiosity-driven ‘blue skies’ research and taxonomy, i.e., basic scientific research with no immediate practical application (Luke et al. 2023).

The most recent comprehensive lists of Italian Ephemeroptera date back to 2003 (Buffagni et al. 2003) and 2009 (Buffagni et al. 2009). However, the latter primarily relied on bibliographic sources, with little to no new field data. Additionally, the only two major publications that comprehensively address the taxonomy of Ephemeroptera as a whole are from 1960 and 1983. In 1960, Grandi published a significant volume reviewing the order (Grandi 1960). In 1983, Belfiore focused on the larval stages of Ephemeroptera in Italy and provided identification keys (Belfiore 1983). In fact, it has been more than 25 years since any research group in Italy has systematically addressed the taxonomy of Ephemeroptera. Nearly a decade ago, with the advent of new molecular biology techniques, Belfiore’s group initiated studies on Italian Ephemeroptera using the barcoding approach to gather data for taxonomic revisions (Cardoni et al. 2015; Tenchini et al. 2018). However, this work was discontinued, and it quickly became evident that the existing taxonomic framework was inadequate and incomplete. Not only were there issues with the incorrect attribution of species names to already known taxa, but it also became clear that several species, which are likely to occur in Italy, have yet to be described.

As part of its Biodiversity Strategy, the EU has enacted the Nature Restoration Law (EU 2024/199), introducing new regulations to achieve “the long-term and sustainable recovery of biodiverse and resilient ecosystems [...] through the restoration of degraded ecosystems.” Member States are required to implement effective, area-based restoration measures to cover at least 20% of the EU’s land area—including freshwater ecosystems—by 2030, and to restore all ecosystems in need of intervention by 2050. However, any strategy aimed at protecting biodiversity or restoring natural areas and ecosystems will be ineffective unless underpinned by robust knowledge of the organisms that form the foundation of biodiversity assessments. Comprehensive understanding of the animal and plant species within a given environment is critical for evaluating its current status, detecting changes driven by global or local factors, and assessing the success of restoration measures. With this in mind, Italy has set up the National Biodiversity Future Center (NBFC: Labra et al. 2024), that is the first National Research and Innovation Center dedicated to biodiversity, funded by the Italian Ministry of University and Research through European Union funds – NextGenerationEU. As part of the NBFC’s initiatives, efforts have been launched to revise the taxonomy of Ephemeroptera using an integrative taxonomy approach that combines traditional morphological methods with molecular biology techniques. These efforts aim to develop a comprehensive systematic revision of the Ephemeroptera species present in Italy, providing an essential foundation for biodiversity research and conservation in the region.

The primary objective of this paper is to provide a complete list of Ephemeroptera species currently believed to occur in Italy, based on traditional morphological methods. Additionally, it aims to offer a brief description of the most interesting or controversial cases related to certain species. This work is intended to serve as a foundation for future revisions and insights derived from an integrative taxonomy approach. In other words, the goal is to establish a “baseline” or “time zero” that will allow for comparison with the results of ongoing and future research on Italian Ephemeroptera. Furthermore, in order to conduct effective revisions using barcoding and other genetic techniques, it is essential to first establish a starting point based on the conclusions reached by experts during the past decades using traditional approaches. This will help to ensure that any new findings are consistent with existing taxonomic knowledge and with the observed distribution of taxa. Given the high proportion of endemic species in Italy (e.g., Stoch 2000) and the current lack of a comprehensive overview of Italian mayflies, the results presented here are expected to be of considerable interest, particularly at continental and Mediterranean scales.

## ﻿Methods

### ﻿Species list and nomenclature

To compile the species list, we relied exclusively on morphological information and descriptions. Reports and potential advancements from molecular biology (e.g., barcoding or metabarcoding) were intentionally excluded unless they aligned with both genetic and morphological approaches, contributed to a morphological description of the collected or reported taxa, and were published by specialists in the order (e.g., Wagner et al. 2017; Yanai et al. 2022).

The species list presented here is primarily based on the foundational work of Buffagni et al. (2003, 2009) and a study on the distribution of Ephemeroptera species published in 2006 (Belfiore 2006). These three publications draw on the existing literature available at the time of their release, as well as on the authors’ direct knowledge of species occurrence and distribution, derived from their examination of material in their collections. This material, which has been expanded with additional collections after 2008, represents more than 50 years of sampling and serves as the basis for the current work. However, this material has not been uniformly studied, leaving many regions of Italy insufficiently explored. For instance, species in the Italian Alps have yet to be thoroughly investigated. Conversely, certain genera in Sardinia and Sicily have received particular attention due to the high levels of endemism observed in these regions, which have drawn significant interest from researchers.

With a few rare exceptions, no indication is given of the extinction risk of the species and its IUCN category. In fact, it is not possible to provide reliable information at this stage, given the obvious need for taxonomic revision.

The book by Bauernfeind and Soldán (2012) on European Ephemeroptera served as the primary reference for nomenclature, including genera and species names. The only exceptions are the Baetidae*Acentrella* Bengtsson, 1912 *Alainites* Waltz, McCafferty & Thomas, 1994, *Nigrobaetis* Novikova & Kluge, 1987, and the Leptophlebiidae*Euthraulus* Barnard, 1932, which are treated here as genera because of their generally accepted and widespread use. For the sake of simplicity, taxonomic affiliation and authority are given for all species in Table 1.

**Table 1.** List of Ephemeroptera species currently believed to be present in Italy based on morphology. The information is given overall for Italy and for each of the six macro-areas defined in the work to describe the distribution patterns of the species. The fourth column indicates whether the species is considered inquirenda, while the sixth column indicates whether it is endemic. At the bottom of the table, the number of species found overall and of endemic species is given, for Italy and for each of the macro-areas. The percentage of endemic species is also given, both in general terms per macro-area, and indicating the percentage of species found only in each of the six macro-areas. The asterisk preceding the species name indicates that the presence of the species must be confirmed, as it has not been found since the catches that allowed it to be described/reported.

#	Family	Species	Species inquirenda	Italy	Endemic	Lowland and hilly areas of the Po basin	the Alps	Northern Apennines	Central-southern Apennines	Sicily	Sardinia
1	Siphlonuridae	*Siphlonuruslacustris* Eaton, 1870		x		x	x	x	x		x
2	Ametropodidae	*Ametropusfragilis* Albarda, 1878		x		x					
3	Baetidae	*Acentrellasinaica* Bogoescu, 1931		x		x		x	x	x	
4		*Alainitesmuticus* (Linnaeus, 1758)		x		x	x	x	x	x	x
5		*Alainitesbengunn* Yanai & Gattolliat, 2022		x	x						x
6		*Baetisalpinus* (F. J. Pictet, 1843)		x		x	x	x	x	x	
7		*Baetisbuceratus* Eaton, 1870		x		x		x	x	x	x
8		*Baetiscyrneus* Thomas & Gazagnes, 1984		x	x				x		x
9		*Baetisfuscatus* (Linnaeus, 1761)		x		x	x	x	x	x	x
10		*Baetisingridae* Thomas & Soldán, 1987		x	x						x
11		*Baetisliebenauae* Keffermüller, 1974		x		x					
12		*Baetislutheri* Müller-Liebenau, 1967		x		x		x	x	x	
13		*Baetismelanonyx* (F. J. Pictet, 1843)		x		x	x	x	x	x	
14		*Baetisnubecularis* Eaton, 1898		x			x				
15		*Baetispavidus* Grandi, 1951		x		x		x	x	x	
16		*Baetisrhodani* (F. J. Pictet, 1843)		x		x	x	x	x	x	x
17		*Baetisvardarensis* Ikonomov, 1962		x		x		x	x		
18		*Baetisvernus* Curtis, 1834		x		x	x	x	x		
19		*Centroptilumluteolum* (O. F. Müller, 1776)		x		x		x	x	x	x
20		*Cloeondipterum* (Linnaeus, 1761)		x		x	x	x	x	x	x
-		*Cloeonlanguidum* Grandi, 1959	x	(x)	(x)	(x)					
-		*Cloeonpraetextum* Bengtsson, 1914	x	(x)		(x)					(x)
21		*Cloeonsimile* Eaton, 1870		x		x		x	x	x	x
22		*Nigrobaetisdigitatus* (Bengtsson, 1912)		x		x		x	x		
23		*Nigrobaetisniger* (Linnaeus, 1761)		x		x					
24		*Procloeonbifidum* (Bengtsson, 1912)		x		x		x	x		x
25		*Procloeoncalabrum (Belfiore & D’Antonio, 1990)		x	x				**x**		
-		*Procloeonforlivense* Grandi, 1964	x	(x)	(x)	(x)					
-		*Procloeonlacustre* (Eaton, 1885)	x	(x)	(x)	(x)					
-		*Procloeonnemorale* (Eaton, 1885)	x	(x)	(x)			(x)			
26		*Procloeonpennulatum* (Eaton, 1870)		x		x		x	x		
27		*Procloeonpulchrum* (Eaton, 1885)		x		x		x	x	x	
28	Oligoneuriidae	*Oligoneuriellarhenana* (Imhoff, 1852)		x		x		x	x		
29	Heptageniidae	*Anaposzebratus* (Hagen, 1864)		x	x						x
30		*Ecdyonurusalpinus* Hefti, Tomka & Zurwerra, 1987		x			x				
31		*Ecdyonurusbelfiorei* Haybach & Thomas, 2001		x	x	x		x	x	x	
32		*Ecdyonurusbellieri* (Hagen, 1860)	x	x	x					x	
33		*Ecdyonuruscorsicus* Esben-Petersen, 1912		x	x						x
34		*Ecdyonurushelveticus* Eaton, 1883		x		x	x	x	x	x	
35		*Ecdyonurusmacani* Thomas & Sowa, 1970		x							
36		*Ecdyonuruspicteti* (Meyer-Dür, 1864)		x			x				
37		*Ecdyonurusruffii* Grandi, 1953		x	x	x					
38		*Ecdyonurusvenosus* (Fabricius, 1775)		x		x	x	x	x	x	
39		*Ecdyonuruszelleri* Eaton, 1885		x			x				
40		*Electrogenabrulini* Wagner, 2017		x	x	x					
41	Heptageniidae	*Electrogenacalabra* Belfiore, 1995		x	x				x		
42		*Electrogenafallax* (Hagen, 1864)		x	x						x
43		*Electrogenagrandiae* (Belfiore, 1981)		x				x	x	x	
44		*Electrogenagridellii* (Grandi, 1953)		x		x					
45		*Electrogenahyblaea* Belfiore, 1994		x	x					x	
46		*Electrogenalateralis* (Curtis, 1834)		x		x	x	x	x	x	
47		*Electrogenalunaris* Belfiore & Scillitani, 1997		x	x			x			
48		*Electrogenaujhelyii* (Sowa, 1981)		x		x					
49		*Epeorusalpicola* (Eaton, 1871)		x			x				
50		*Epeorusassimilis* Eaton, 1885		x			x	x	x	x	
51		*Epeorusyougoslavicus* (Šamal, 1935)		x					x	x	
52		*Heptageniacoerulans* Rostock, 1878		x		x			x		
53		*Heptagenialongicauda* (Stephens, 1836)		x		x		x	x		
54		*Heptageniasulphurea* (O. F. Müller, 1776)		x		x					
55		*Rhithrogenaadrianae* Belfiore, 1983		x	x				x		
56		*Rhithrogenaalpestris* Eaton, 1885		x			x				
57		*Rhithrogenadegrangei* Sowa, 1969		x			x				
58		*Rhithrogenadorieri* Sowa, 1971		x			x				
59		*Rhithrogenafiorii* Grandi, 1953		x	x			x	x		
60		*Rhithrogenahybrida* Eaton, 1885		x			x	x	x		
61		*Rhithrogenajohannis* Belfiore, 1990		x	x				x	x	
62		*Rhithrogenaloyolaea* Navás, 1922		x			x	x	x		
63		*Rhithrogenanivata* (Eaton, 1871)		x			x				
64		*Rhithrogenanuragica* Belfiore, 1987		x	x						x
65		*Rhithrogenareatina* Sowa & Belfiore, 1984		x	x				x		
66		*Rhithrogenasavoiensis* Alba-Tercedor & Sowa, 1987		x				x			
67		*Rhithrogenasemicolorata* (Curtis, 1834)		x		x	x	x	x	x	
68		*Rhithrogenasiciliana Braasch, 1989	x	x	x					**x**	
69	Leptophlebiidae	*Choroterpesborbonica* Belfiore, 1988		x	x				x	x	
70		*Choroterpespicteti* (Eaton, 1871)		x		x		x	x		
71		*Euthraulusbalcanicus* Ikonomov, 1961	x	x		x					
72		*Habroleptoidesauberti* Biancheri, 1954		x			x				
73		*Habroleptoidesconfusa* Sartori & Jacob, 1986		x		x	x	x	x		
74		*Habroleptoidesmodesta* (Hagen, 1864)		x	x						x
75		*Habroleptoidespauliana* (Grandi, 1959)		x	x			x		x	
76		*Habroleptoidesumbratilis* (Eaton, 1884)		x	x			x	x		
77		*Habrophlebiaconsiglioi* Biancheri, 1959		x	x						x
78		*Habrophlebiaeldae* Jacob & Sartori, 1984		x		x		x	x	x	x
79		*Habrophlebiafusca* (Curtis, 1834)		x		x		x			
80		*Habrophlebialauta* Eaton, 1884		x		x		x			
81		*Paraleptophlebiaruffoi* Biancheri, 1956		x	x	x			x		
82		*Paraleptophlebiasubmarginata* (Stephens, 1836)		x		x		x	x		
83		*Thraulusbellus* Eaton, 1881		x		x		x			
84	Ephemeridae	*Ephemeradanica* Müller, 1764		x		x	x	x	x		
85		*Ephemeraglaucops* Pictet, 1843		x		x		x	x		
86		*Ephemeravulgata* Linnaeus, 1758		x		x					
87		*Ephemerazettana* Kimmins, 1937		x		x					
88	Polymitarcyidae	*Ephoron virgo* (Olivier, 1791)		x		x		x	x		
89	Potamanthidae	*Potamanthusluteus* (Linnaeus, 1767)		x		x		x			
90	Ephemerellidae	*Ephemerellaignita* (Poda, 1761)		x		x	x	x	x	x	x
91		*Ephemerellamucronata* (Bengtsson, 1909)		x		x					
92		*Serratellaikonomovi* (Puthz, 1971)		x					x	x	
93		*Torleyamajor* (Klapálek, 1905)		x		x		x			
94	Caenidae	*Brachycercusharrisellus* Curtis, 1834		x		x		x			
95		*Caenisbelfiorei* Malzacher, 1986		x	x				x		
96		*Caenisbeskidensis* Sowa, 1973		x		x		x			
97		*Caenishoraria* (Linnaeus, 1758)		x		x	x	x			
98		*Caenislactea* (Burmeister, 1839)		x		x					
99		*Caenisluctuosa* (Burmeister, 1839)		x		x		x	x	x	x
100		*Caenismacrura* Stephens, 1836		x		x					
101		*Caenismartae* Belfiore, 1984		x	x			x	x	x	x
102		*Caenispseudorivulorum* Keffermüller, 1960		x		x		x	x		
103		*Caenispusilla* Navás, 1913		x		x		x	x	x	
104		*Caenisrobusta* Eaton, 1884		x		x					x
105		*Caenisvalentinae Grandi, 1951		x	x	x					
106	Prosopistomatidae	*Prosopistomapennigerum (O. F. Müller, 1785)		x				x?			
		Number of species		106	29	63	29	55	56	33	23
		Number of endemic species				5	0	6	13	6	10
		% endemic species (Italy and Med)			27.4	7.9	0.0	10.9	23.2	18.2	43.5
		No. endemic species present only in one area				3	0	1	5	3	8
		% endemic species present only in one area				4.8	0.0	1.8	8.9	9.1	34.8

Given the importance of establishing verifiable references for the identification of species present in Italy, a specimen vouchering process supported by COI barcoding (AB, unpublished) was initiated as part of the ITINERIS project, with best-practice procedures for archiving of selected Ephemeroptera based on GBIF and Darwin Core standards.

### ﻿Outline of species distribution

At least two primary needs can be identified when drafting species distributions. The first is to provide highly detailed information about the specific areas and locations where species have been found. The second is to identify and summarise broader distribution patterns, providing a general overview and allowing comparison and correlation of data across species. Due to the absence of a centralised repository for Ephemeroptera distribution data and the resulting gaps in detailed knowledge for many areas, the second approach has traditionally been preferred. In Buffagni et al. (2009), a book focusing on European Ephemeroptera, species distribution is presented according to Illies’ ecoregions (Illies 1978). For Italy, this framework includes Ecoregion 3 (Italy), encompassing Sardinia and Sicily, and Ecoregion 4, corresponding to the Alps. In earlier checklist efforts for Italian fauna (i.e., Minelli et al. 1993, 1995; Stoch and Minelli 2004), Italy was instead divided into two macro-areas, North and South, with the fauna of Sardinia and Sicily treated separately. Taking a contrasting approach, the ckMAP project (Ruffo and Stoch 2006) presented the distribution of numerous Italian species, including Ephemeroptera (Belfiore 2006), using geographic quadrants of approximately 10 × 10 km. However, much of the data used to determine these distributions—drawn from available literature and a taxonomy that has since undergone extensive revisions at the European level—would now likely require significant updating. There is therefore little point in undertaking such a review of species distributions at this time, which could instead be one of the objectives of ongoing activities, with a time horizon of 5–10 years.

**Figure 1.**
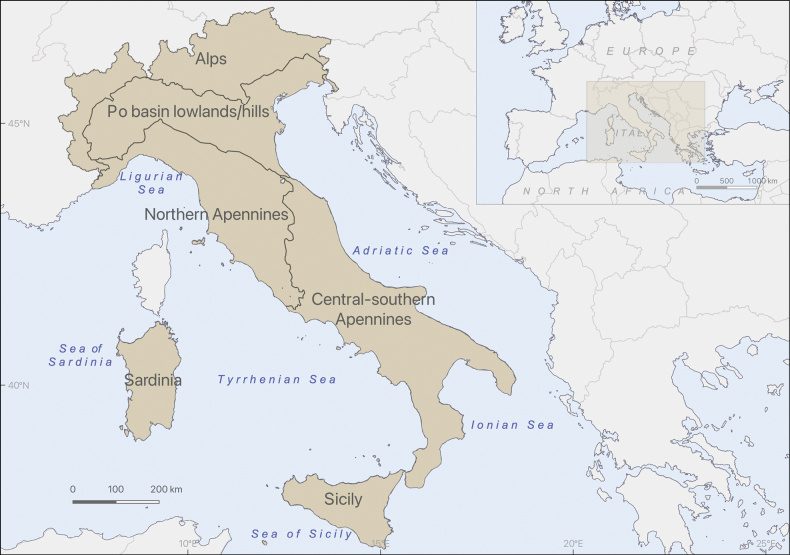
Map of the six macro-areas used in the work to describe the distribution of Ephemeroptera species in Italy. Apart from the delimitation of Sardinia and Sicily, the other limits must be more properly considered as bands.

For this work, we opted for an intermediate scale, dividing the Italian territory into six main areas. These are: the Alps, the lowland and hilly areas of the Po basin (≈ < 300 m, including the lowlands of Friuli-Venezia Giulia and the lower Adige catchment), the northern Apennines, the central-southern Apennines, Sardinia, and Sicily (Fig. 1).

The distinction between the Alps/Apennines and the lowland/hilly areas of the Po basin is based on ecological factors that influence species distribution. More specifically, to define the boundary between the lowland/hilly areas and the mountain areas, we used the boundaries already defined for hydro-ecoregions (Chandesris et al. 2006; Wasson et al. 2006). Hydro-ecoregions (HERs) are officially used in the Italian river typology (see Suppl. material 1 and: Buffagni et al. 2006; MATTM 2008: Annex I, fig. 1.1) for the Water Framework Directive (WFD), also for the assessment of ecological status based on benthic macroinvertebrates. Hydro-ecoregions 5, 6, 7 (Italian part), and 8 together make up the part of the territory referred to here as the lowland and hilly areas of the Po basin (see Annex 1). Sardinia and Sicily are treated separately due to the high number of endemic species on these islands. The division of the Apennines into northern and central-southern zones roughly reflects a discontinuity in Ephemeroptera distribution previously identified by Belfiore (1994a). For convenience and consistency with the data collected under the WFD, this boundary has been aligned with the division between HERs 10, 11, 14, and HERs 12, 13, 15 (Annex 1). In any case, with the exception of Sardinia and Sicily, these zones are approximations. Actually, the dividing lines shown in Fig. 1 should be interpreted as broad bands rather than precise boundaries, as the current knowledge of species distribution is not detailed enough to accurately define the separations between these areas.

## ﻿Results

### ﻿The big picture

Table 1 provides a comprehensive list of Ephemeroptera species currently believed to be present in Italy, grouped by family according to the classification system outlined by Bauernfeind and Soldán (2012). For each of the six previously described macro-areas, the table indicates the possible presence of each species. Additionally, it notes whether a species is endemic to Italy.

In total, we report the presence of 106 species of Ephemeroptera in Italy. Of these, 29 species are endemic, accounting for approximately 27% of the total. Among the six macro-areas, the Po Valley hosts the highest number of species, with 63 recorded, followed by the central-southern and northern Apennines, reporting 56 and 55 species, respectively. Approximately 30 species are reported for both Sicily and the Alpine region, while Sardinia has the lowest total, with just 23 species.

An examination of the number of species endemic to Italy or southern Europe by macro-area reveals interesting patterns. In the Po Valley, only five endemic species are recorded, likely reflecting the overlap of species common to other European regions. The northern Apennines show ~ 11% endemic species. Endemism rises significantly in Sicily, where it exceeds 18%, and in the central-southern Apennines, where it reaches 23%. Sardinia, despite hosting the fewest species overall, exhibits a remarkably high level of endemism, with > 40% of its species classified as endemic.

Focusing on endemic species that are unique to a single area (i.e., present in only one of the six macro-areas adopted here to present species distributions) provides further insights. Overall, as many as 20 of the 29 endemic species believed to be present in Italy are found in only one of the six macro-areas. In the Po Valley and the northern Apennines, the proportion of species endemic to these regions remains < 5%. This figure increases to nearly 10% in the central-southern Apennines and Sicily. Sardinia stands out with nearly 35% of its species being endemic solely to the region (including Corsica), making it the richest biodiversity hotspot for endemic mayflies in Italy. The high levels of endemism observed in areas such as Sardinia and Sicily suggest the potential for undiscovered species, particularly in Sicily, where additional endemic Ephemeroptera may still await description. In fact, to date not all families/genera have been studied with the same emphasis. These findings underscore the importance of targeted biodiversity studies to further refine our understanding of Italian mayfly diversity and endemism.

According to Bauernfeind and Soldán (2012), eight of the taxa listed here are classified as species inquirenda, meaning that the available knowledge is insufficient to confirm their validity with certainty. Consequently, data regarding their presence and distribution should be interpreted with caution. These taxa are not included in the calculation of species present in Italy, except for three species discussed in the text. A subset of these species has not been recognised or reported—if collected—since their initial taxonomic description. Additional information on these taxa is provided in the following paragraphs.

Since the most recent publication providing synoptic information on Italian Ephemeroptera (Buffagni et al. 2009), two new species have been described based on material collected in Italy: *Electrogenabrulini* Wagner, 2017, identified in small streams in the hilly regions of Lombardy (Wagner et al. 2017), and *Alainitesbengunn* Yanai & Gattolliat, 2022, discovered in Sardinia (Yanai et al. 2022).

### ﻿Results and comments on mayfly species

The following section provides commentary on some of the mayfly species found in Italy, aiming to highlight potential issues or critical situations of various kinds. For the four most represented families—Heptageniidae, Baetidae, Leptophlebiidae, and Caenidae—the species-specific comments are grouped within the same paragraph for clarity and coherence. With a few exceptions, we have not made a comparison with the distribution of species in neighbouring countries. Since the Ephemeroptera fauna of these countries is usually better known than that of Italy, we do not believe that such a comparison would have been particularly valuable, especially at this stage of taxonomic revision in Italy. Moreover, such an activity would have implied a different approach to the work, which is not in line with the proposed objectives.

### ﻿The special case of *Ametropusfragilis* Albarda, 1878

The situation of *Ametropusfragilis* is discussed in greater detail than other species because it highlights some interesting and potentially generalisable challenges regarding the detection and actual presence of rare taxa in Italy.

In the recent history of Ephemeroptera research, *Ametropusfragilis* was first collected in the lower stretch of the Adige River during the winter of 1993–1994 (Turin et al. 1997), marking the species’ first documented occurrence in Italy. Its presence was initially attributed to a possible accidental introduction through fish restocking practices involving stocks imported from Eastern European countries. However, a subsequent study by Sartori and Bauernfeind (2020) revealed that an adult male of this species, collected in Italy during the 19^th^ century, is preserved in the Pictet collection at the Geneva Museum. Although Pictet correctly identified the specimen, he did not report the species’ presence in Italy (Sartori and Bauernfeind 2020). This evidence suggests that *Ametropusfragilis* has historically occurred in Italy but was not observed again until its rediscovery in 1993 (Turin et al. 1997). This more recent collection occurred during a biomonitoring activity for which a specific identification for mayflies was not expected (Turin et al. 1997). The genus *Ametropus* was not reported in Italy at the time, making the discovery appear “odd” and prompting further investigation. It is plausible that if the species had belonged to a multi-species genus already documented in Italy, it might have gone unnoticed. Further discoveries, such as the first collection of the species in Croatia (Ćuk et al. 2015) and other neighbouring countries, combined with its apparent survival in Italy for > 150 years without detection, underscore the need for targeted research in suitable habitats. These include large rivers with sandy or muddy substrates and the presence of organic detritus.

The fragmented history of *Ametropusfragilis* carries important implications. For certain species, extinction may be only apparent, and populations may persist undetected for decades in specific conditions. For example, nymphs of *A.fragilis* were recorded for the first time in the Ipeľ (Ipoly) river on the Slovak-Hungarian border (Macko and Derka 2021), although the river had previously been studied relatively intensively by authors from both sides of the border (Macko et al. 2023). The rediscovery of *Ametropusfragilis*, a species that is relatively easy to identify, raises the possibility that similar scenarios could exist for more cryptic or less distinctive species. These might go unnoticed during routine sampling or standard biomonitoring protocols. In addition, the combination of habitat fragmentation in rivers and the low population densities exhibited by many Ephemeroptera species underscores the importance of targeted sampling efforts. Such efforts should aim to identify faunal “emergencies” and contribute to a more accurate picture of overall biodiversity.

### ﻿Baetidae species

The taxonomic status within the family Baetidae is both complex and dynamic. Molecular methods have revealed the existence of numerous putative and/or undescribed species across various genera and species groups (Cardoni et al. 2015; Tenchini et al. 2018; AB unpublished). For instance, several species are collectively identified under the name *Alainitesmuticus* (see Yanai et al. 2022), as well as *Baetisingridae*. Regarding the latter, molecular techniques have demonstrated the presence of three distinct species from the *rhodani* group in Sardinia (Bisconti et al. 2016). Similarly, molecular biology and barcoding techniques have clarified that the taxon previously referred to as *Baetisfuscatus* actually encompasses a species complex (Cardoni et al. 2015; El Yaagoubi et al. 2023), with many of these species awaiting formal description.

*Baetispavidus*, was once prevalent and relatively abundant in the Po Valley. However, during the past decade, its distribution in northern Italy, including its type locality, has declined sharply—likely as a consequence of climate change—with no recent specimens available from this region. The species has also nearly vanished from central Italy. In contrast, some specimens collected in Sicily have been identified as presumably belonging to this species.

*Procloeonlacustre* (Eaton, 1885) and *Procloeonnemorale* (Eaton, 1885), belonging to the subgenus Pseudocentroptilum Bogoescu, are not included in the list of Italian mayflies because their status is essentially unclear and we have never directly examined material of these species. Both species were described by Eaton in 1885 and have not been reported since. Similarly, *Procloeonforlivense* (Grandi, 1964) and *Cloeonlanguidum* Grandi, 1959, although described from Italy by an Italian author, are not included in the list. These four species, together with *Cloeonpraetextum* Bengtsson, 1914, are considered as *species inquirenda* by Bauernfeind and Soldán (2012). There are more species of these genera, and perhaps of *Centroptilum* Eaton, in Italy than are included in the checklist. However, at present we do not have any conclusive elements to clarify the picture regarding these interesting genera.

### ﻿Heptageniidae species

Alongside the genus *Anapos* Yanai & Sartori, 2017, represented by a single species in Sardinia, the only other genus in this family with a reasonably accurate understanding of its species composition is *Electrogena* Zurwerra & Tomka, 1985. This genus has been the focus of extensive research over the years (e.g., Belfiore 1994b, 1995, Belfiore et al. 1997) and a comprehensive review (Belfiore 1996). For other genera, both species-poor ones like *Heptagenia* Walsh, 1863 and *Epeorus* Eaton, 1881 and species-rich ones such as *Ecdyonurus* Eaton, 1868 and *Rhithrogena* Eaton, 1881, significant changes are expected in the near future. These changes will likely stem from the discovery of previously unreported species and the formal description of new taxa. Indeed, morphological analyses of collected material, supported by ongoing genetic studies (AB unpublished), have revealed the presence of taxonomic entities that cannot yet be confidently assigned to any currently known species.

As far as the taxa of the genus *Ecdyonurus* present in Italy are concerned, a few species are easily recognised in both adult and larval stages and have a clear status i.e., *E.alpinus*, *E.belfiorei*, and *E.corsicus*, while for all other species taxonomic refinements with the selection of new diagnostic characters would be appropriate. In many cases, a careful analysis of the distributional ranges of different taxa is likely to be an effective aid in interpreting the taxa actually present, making it possible to highlight any discontinuities or gradients in intraspecific morphological variability.

As far as the *Rhithrogena* genus, we report here the presence of *R.savoiensis*, which is new for Italy (upstream reaches of river Marecchia, 22.6.2019, C. Belfiore leg. det.). In general, a few species of the genus have a clear status i.e., *R.adrianae*, *R.johannis*, *R.nivata*, *R.nuragica*, *R.reatina*, and *R.savoiensis*. For all the other species, the specific name and attribution should be verified. Consequently, all information available to date on the distribution and ecology of the various species of the genus reported for Italy must be considered with great caution, given the inherent degree of uncertainty of the specific identification itself. According to Tenchini et al. (2018), *Rhithrogenasibillina* Metzler, Tomka & Zurwerra, 1985 is considered synonymous with *Rhithrogenareatina* Sowa & Belfiore, 1984 and is therefore not reported in Table 1.

### ﻿Leptophlebiidae species

With regard to this family, while being aware that identification at the larval stage is sometimes based on characters that should be reviewed, the situation- compared to other families- seems clearer. For the status of two species, *Habroleptoidesmodesta* (Hagen, 1864) and *Habroleptoidesumbratilis* (Eaton, 1884), clarification would be appropriate in general, not only for Italy. *Paraleptophlebiaruffoi* Biancheri, 1956 and *Habroleptoidesauberti* (Biancheri, 1954) would require further investigation regarding their distribution. The taxon *Euthraulusbalcanicus* Ikonomov, 1961, is clearly distinct from the related species found in Italy, although it is considered a *species inquirenda* (Bauernfeind and Soldán 2012).

### ﻿Caenidae species

In general, there are probably more species in the *macrura* group than those currently listed. However, the overall situation and the potential for describing new species is complicated by the high morphological similarity between the taxa, especially in the larval stages, which makes it difficult to highlight the species really present in Italy. A similar argument to that made for the Baetidae, i.e., that some species would be more appropriately considered as groups of species, probably applies to some species of Caenidae. For example, it is very likely that *Caenismartae* Belfiore, 1984 comprises several cryptic species occurring in different parts of Italy, sometimes even in sympatry.

*Caenisbelfiorei* Malzacher, 1986 is widespread in southern Italy, where it is sympatric with *C. pseudorivulorum* Keffermüller, 1960. *Caenisbelfiorei* was originally described as a subspecies of *C. pseudorivulorum* but, according to Buffagni (1997, 1999), it is elevated to the species status. Another species of the *pseudorivulorum* group is present in Italy, *C. beskidensis* Sowa, 1973, which seems to be restricted to the northern Apennines.

### ﻿Other species and further comments

Across the different families of Ephemeroptera, we have a number of easily identified species whose presence in Italian watercourses seems to have decreased considerably in the last two decades. Although this indication results mainly from the direct activity of the authors not expressly aimed at assessing the range of these species and from occasional analyses of biomonitoring data, it unfortunately seems that the presence of the species is rarer than in the past, although some of them may locally reach high densities where present. Among these species, mention should be made of *Ephoron virgo* (Olivier, 1791), *Oligoneuriellarhenana* (Imhoff, 1852), *Acentrellasinaica* Bogoescu, 1931, *Heptageniacoerulans* Rostock, 1878 and *Nigrobaetisdigitatus* (Bengtsson, 1912).

Then there are other, relatively rare species whose distribution in Italy has never been really known in detail and which would require specific surveys to confirm their presence and verify the conservation status of their habitats. These include *Ephemeravulgata* Linnaeus, 1758, *Ephemeraglaucops* Pictet, 1843, *Nigrobaetisniger* (Linnaeus, 1761), *Ecdyonuruszelleri* (Eaton, 1885), *Ecdyonurusruffii* Grandi, 1953, *Rhithrogenanivata* (Eaton, 1871), *Caenisbeskidensis* Sowa, 1973, and *Caenislactea* (Burmeister, 1839).

Finally, we have five species no longer found after the first collection, which, in four cases, supported the species description. A very interesting and peculiar species, both for its morphology and for the environments it colonises (Schletterer and Füreder 2009), is *Prosopistomapennigerum* (Müller, 1785), reported only once from Tuscany, with observations made in 1979 (Bellmann, 2000; Schletterer and Füreder 2009). The species, due to the profound alterations suffered by the environment in which it was collected (i.e., the final stretch of the River Ombrone) is probably extinct.

Although we have not directly analysed specimens of the two species, *Ecdyonurusbellieri* (Hagen, 1860) and *Rhithrogenasiciliana* Braasch, 1989, which were no longer collected after their description, they are presented in the taxa list. In this case, although they are both *species inquirenda*, they were described on material collected in Sicily, a land rich in endemism, and we have, therefore, cautiously preferred to record them in the taxa list.

*Caenisvalentinae* Grandi, 1951 and *Procloeoncalabrum* (Belfiore and D’Antonio, 1990), Italian endemics, have not been caught since the collections that allowed their description. Dedicated sampling campaigns are planned in the near future in the hope of confirming the presence of these two species and thus their non-extinction.

## ﻿Discussion

### ﻿Basic deficiencies in the Italian Ephemeroptera and new criticalities

The general picture of the Italian Ephemeroptera, briefly described above, allows us to highlight some relevant aspects to guide future research on the taxonomy of this order. A first element, at once of great stimulus for research and of concern, is that several species described on material collected in Italy have not been caught since the initial collection. In some cases, type material appears to have been lost or, where present, is not usable for genetic analysis. The collection of new material from type localities is therefore urgent. Some species are known from only one or a few close localities. In this case, new collections are indispensable, although there is no guarantee of finding the species again. When a species has been described from specimens collected from different areas in Italy, it will be easier to make new collections, but it will be advisable to check whether all specimens belong to a single species, possibly with the aid of genetic techniques, for the possible presence of cryptic species. Consequently, such verification should also be done for possible lectotypes or syntypes, whenever possible.

As mentioned above, there are currently insufficient data for the compilation of a Red List of Italian Ephemeroptera, although this is a matter of extreme urgency. At present, there is no repository for information on the collection of Ephemeroptera species and, in fact, data are archived, in a more or less systematic and organised manner, to manage the authors’ physical collections. At the national level, there are repositories of information at genus or family level. In general, apart from the activities of the authors, few attempts exist in Italy to arrive at a specific identification of the Ephemeroptera.

The focus on Ephemeroptera has traditionally been on running water environments, which are home to many more species than lentic environments. This has therefore led to a lack of knowledge about the species present in ponds and lakes. In any case, the greatest urgency for further knowledge seems to be related to lotic environments, by virtue of their tendency to become temporary (Skoulikidis et al. 2017), with changing community structure and the potential replacement of species from more lotic to more typically lentic (Buffagni 2021). Urgency is particularly linked to global changes, where climate change has exacerbated an already critical situation in some areas and ecosystem categories (Alba-Tercedor et al. 2017). Habitat changes and alterations affect the occurrence and distribution of mayflies and can also affect rare and endemic species (e.g., Buffagni et al. 2016). Similarly, climate change may pose further difficulties in planning focused collections due to the unpredictability of river flows, particularly in the Mediterranean, and may alter expected reference conditions (De Girolamo et al. 2017).

The advent of new genetic techniques and standards for comparing different taxa and defining the boundary between different species can facilitate taxonomic investigation (Yeates et al. 2011). Integrative taxonomy (e.g., Schlick-Steiner et al. 2010) has become an essential tool for taxonomic revisions, including that of Ephemeroptera. In Italy, a systematic barcoding initiative for Ephemeroptera is underway (AB unpublished) and is anticipated to clarify the broader taxonomic framework of major families within a few years. This effort is expected to significantly accelerate the study of Ephemeroptera, enabling a more rapid determination of the number and diversity of taxonomic entities present in Italy. However, challenges will persist in interpreting the identified clades. These must be cross-referenced with their geographical distribution and, crucially, with the morphological characteristics of the collected specimens (e.g., Stoch et al. 2024). Simply recognising clades—or in some cases, defining putative species (e.g., Bisconti et al. 2016)—will not necessarily conclude the taxonomic process, which ideally culminates in the formal description of the identified species. Achieving this will require the involvement of highly skilled researchers proficient not only in genetic methods but also in detailed morphological analysis.

### ﻿Lack of recent taxonomic research: reasons

The primary reason for the limited focus on taxonomic studies of Ephemeroptera appears to be the lack of dedicated funding (reason 1). The scarcity of resources for basic research, combined with a broader shift towards more applied scientific topics (reason 2), has rendered taxonomic studies increasingly unattractive, uninteresting, or simply unsustainable for newer generations of researchers in Italy (see Kim 2017, for general considerations on the moribund state of insect taxonomy). This issue will be briefly addressed below. Here, however, we aim to concentrate on the epistemological and gnoseological dimensions of the topic.

Although accurate and reproducible taxonomic identification is the cornerstone of biology, the level of validation of taxonomic identification in entomological studies is often poor (Packer et al. 2018). Notably, since the mid-1990s, no Italian researchers have systematically committed to studying the taxonomy of Ephemeroptera with the goal of addressing the numerous poorly understood aspects of the species present in Italy. Essentially, the principle that inaccurate information could lead to subsequent interpretative errors (reason 3; e.g., Bortolus 2008) has significantly hindered major taxonomic advancements in Italian mayflies for at least two decades. This perception arises from the difficulty of detecting and correcting such inaccuracies solely through the literature. As a result, when identification errors are anticipated, tentative species-level identifications often contribute minimally to the advancement of knowledge while introducing potentially enduring and harmful biases over time. The literature is replete with examples of species names being inaccurately assigned due to limited understanding of taxonomic order, erroneous assumptions about species distribution, or inadequate species definitions by specialists. This issue is partly a consequence of minimal or non-existent funding for taxonomic research—an ineffective strategy that has affected not only Ephemeroptera but a broad range of aquatic taxa. Such a short-sighted approach has also caused lasting harm to research opportunities, restricting the potential for progress in these fields. For example, the lack of resources—particularly preservation tools, infrastructures and dedicated personnel (reason 4; e.g., Engel et al. 2021)—to establish and maintain natural history collections has led to the loss or alteration of many collected mayfly specimens. Frequently, re-examining original material or preserved specimens proves difficult, if not impossible. These specimens are often fragmented or partially damaged during initial microscopic analyses, and many have been entirely lost over the decades (reason 5). This situation severely hampers the application of the scientific method, limiting the ability to refute or confirm historical (and published) findings. This challenge is particularly evident for numerous species, including potential endemics, described during the 19^th^ century or the mid-20^th^ century. The task of rediscovering and recollecting species found decades ago—often poorly described—within a natural environment that has undergone significant changes, coupled with the necessity of integrating different methodological approaches (e.g., genetics and morphology), makes this undertaking exceptionally demanding.

### ﻿Lack of recent taxonomic research: consequences and perspectives

Comparative tables and the quantification of diagnostic characteristics for species identification and divergence—along with the species descriptions and reviews compiled by the authors—are likely outdated. These are often based on a limited selection of species present in Italy (and Europe), as recent biomolecular analyses increasingly indicate. This suggests that interspecific differences may have been conflated with intraspecific variability, or that inappropriate diagnostic characteristics were selected. For example, *Ecdyonurusaustriacus* was not recorded in Italy when the review of Italian species in the *helveticus* group was compiled (Belfiore and Buffagni 1994), but its presence is now considered highly likely. Thus, it is possible that some of the nymphs previously attributed to *E.alpinus* may actually belong to *E.austriacus*. A similar scenario could apply to *Caenismartae*, whose purported distribution and morphological variability suggest the potential coexistence of similar, closely related species in Italy. As mentioned above, a further examination of the material used for taxonomic reviews is needed to confirm or refute the identifications and the assignment of quantitative thresholds to diagnostic characters. In practice, this task appears challenging, and new specimens are currently being collected for refinement. However, such work should be conducted for most species groups of Italian mayflies, requiring significant effort and dedicated research. Will it be possible to find/form experts available to complete such a time consuming work that is – additionally – not that much appreciated by scientific evaluation boards (e.g., too low Impact Factor: Agnarsson and Kuntner 2007)? Most of the Ephemeroptera books cited in the introduction of this manuscript (e.g., Grandi 1960; Belfiore 1983; Bauernfeind and Soldan 2012; Buffagni et al. 2009) took months or years of work to be written and are based on skills acquired over years or decades. None of them even have an Impact Factor: in different historical and research periods and contexts, their value is, however, indisputable. Nowadays, there is the believe that fundamental (vs. applied) research has a great value. In the meantime, innovative ideas, conceptions and papers are encouraged, pushing quality of research (e.g., few papers targeting Nature or Science) instead of quantity (more papers of ‘lower’ level). The description of ‘new’ species – we still expect many in Europe – is *per se* innovative and, of course, intrinsically a piece of fundamental research, which is crucial for understanding, managing and teaching biodiversity (Kim 2017; Härtel et al. 2023). Understanding local species and incorporating their study into environmental education should be central to fostering a deeper interest in the natural environment, particularly among younger generations (Genovart et al. 2013). Nevertheless, the description of new species, although at the heart of the study of biodiversity, is confined to a small number of specialist publications, with Impact Factors significantly lower than most journals devoted to more applied research. The description of new species- if not perhaps of a new Hominidae- is unlikely to find a place in the more emblazoned journals. In the hope that the world of research will find a balance between the performance needs of young researchers, environmental urgencies and the need to quantify and protect biodiversity, Italy is now offering a perspective for new generations with the creation of the National Biodiversity Future Centre. NBFC organises PhD courses on biodiversity and taxonomy, to encourage, instruct and accompany young researchers on the long way of taxonomic investigation. Hopefully, some effort will be devoted to aquatic organisms, too, beyond the minimal scheduled life of the Centre (2022–2025).

## ﻿Concluding thoughts

This paper aims to contribute meaningfully to the study of Italian Ephemeroptera, acknowledging both the highly dynamic nature of species concepts and the importance of incremental advances in scientific understanding. We argue that morphospecies (i.e., an approximate typological interpretation of Linnaean species) hold a crucial role in establishing ‘stable’ reference points (Sandall et al. 2023). These reference points, in use for centuries, are essential for enabling effective information exchange and fostering communication with a wider audience (e.g., Lindemann-Matthies and Bose 2008). This encompasses foundational efforts, such as educating children (Randler 2008), and addressing the tendency for invertebrates to be overlooked compared to vertebrates and plants (Leather and Quicke 2010; Mammola et al. 2023). Molecular biology and genomic approaches will enable further in-depth studies, likely leading to progressive fragmentation of taxa into dynamic entities that vary across spatial and temporal scales and/or with speciation driven by introgression of ecological traits (Rosser et al. 2024). The feasibility of describing morphospecies that align with genetic entities will depend on several factors, including the degree of taxonomic diversification within genera and species groups, the presence of sister and/or cryptic species, the extent of investigative efforts, and available funding. Consequently, morphospecies (and their associated names) are likely to correspond to genetically derived taxonomic units and categories that vary across different species groups and taxa (Sbordoni 2010). Species description and ‘naming’ remain powerful tools for characterising and quantifying biodiversity, particularly when targeting communication efforts towards the general public, citizens, institutions, and students of school age. Contrary to the trends observed in Italy during the past 30 years, it seems timely and appropriate to redirect attention toward the description of new Ephemeroptera species, ensuring these efforts are supported by detailed morphological analyses (Huber et al. 2024). Integrative taxonomy, which combines COI sequences with morphological data (Hubert and Hanner 2016), should be embraced to improve the reliability of species identification. However, overly concise morphological descriptions, as seen in turbo-taxonomy practices (Butcher et al. 2012), should be avoided in the case of Italian mayflies. This approach ensures that species remain recognisable not only to specialists but also to a broader audience, without relying solely on genetic analyses.

In closing, we wish to highlight one of the apparent paradoxes of biodiversity. In a world where extinction rates are alarmingly high, the paradox lies in the fact that the number of recorded species may appear to increase simply because these species- which in reality are likely to have existed for millennia- are defined and described by the academic community (Blackburn et al. 2019) or because colonisations are detected earlier than extinctions (e.g., Kuczynski et al. 2023). This poses a genuine risk that non-specialists and policymakers might mistake this growth in knowledge—primarily driven by methodological advancements in the discovery of new species—and improved communication, for an improbable increase in actual biodiversity.

## ﻿Additional information

### Author ORCIDs

Andrea Buffagni https://orcid.org/0000-0002-3944-1861

Carlo Belfiore https://orcid.org/0000-0002-0061-8658

### Data availability

All of the data that support the findings of this study are available in the main text or Supplementary Information.

## Supplementary materials

Supplementary material 1 Map of the Italian Hydro-ecoregions Data type pdf https://binary.pensoft.net/file/1340179This dataset is made available under the Open Database License (http://opendatacommons.org/licenses/odbl/1.0/). The Open Database License (ODbL) is a license agreement intended to allow users to freely share, modify, and use this Dataset while maintaining this same freedom for others, provided that the original source and author(s) are credited.Andrea Buffagni, Carlo Belfiore
