# Supplementary material for: ﻿Biodiversity of Italian freshwaters: an updated checklist of mayfly species (Ephemeroptera) as a starting point for the next taxonomic (r)evolution
Source: Zookeys. 2025 May 28;1239:257–80. doi: 10.3897/zookeys.1239.147826 (PMC12138380; doi:10.3897/zookeys.1239.147826)
Supplement: Supplementary material 1 — Map of the Italian Hydro-ecoregions [file zookeys-1239-257_article-147826__-s001.pdf]

Biodiversity of Italian freshwaters: an updated checklist of mayfly species (Ephemeroptera) as a starting point for the next taxonomic (r)evolution.

ZooKeys

Andrea Buffagni* & Carlo Belfiore

*corresponding author - CNR-IRSA, National Research Council, Water Research Institute, Via del Mulino 19, 20871 Brugherio (MB), Italy, andrea.buffagni@irsa.cnr.it

Supplementary Information

Map of the Hydro-ecoregions (HERs) officially in use for the Italian river typology (Buffagni et al., 2006; MATTM, 2008: Annex I, Fig 1.1) of the Water Framework Directive (WFD). HERs are also used to assess ecological status based on benthic macroinvertebrates. HERs are coded with a number between 1 and 21. The names of each HER are placed on the map. The boundaries between the six macro-areas used in the work to describe the distribution of Ephemeroptera species trace the boundaries between some of these HERs. Hydro-ecoregions 5, 6, 7 (Italian part) and 8 together make up the part of the territory referred to here as the lowland and hilly areas of the Po basin. The division of the Apennines into northern and central-southern zones is aligned with the division between HERs 10, 11, 14, and HERs 12, 13, 15.

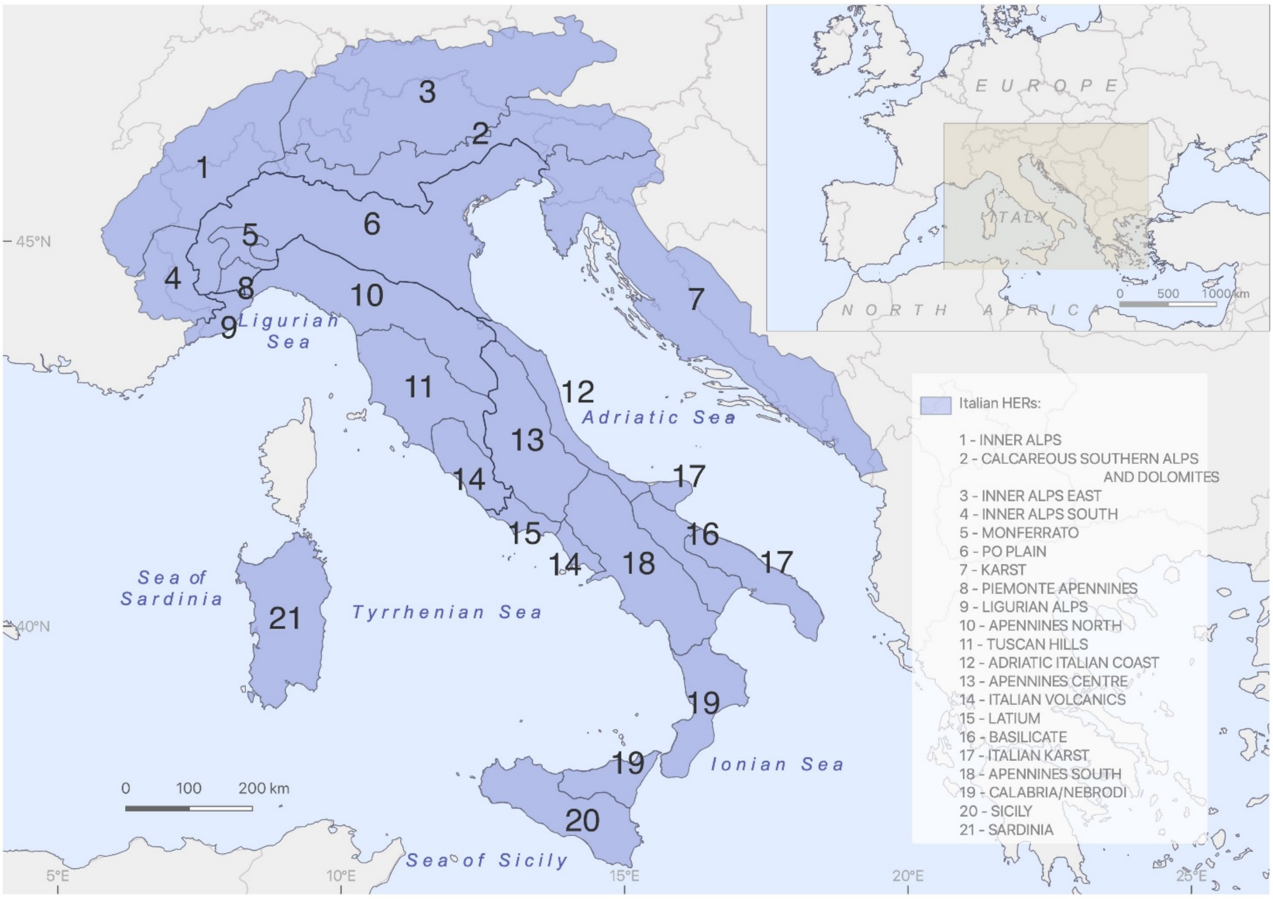

Buffagni, A., M. Munafò, F. Tornatore, I. Bonamini, A. Didomenicantonio, L. Mancini, A. Martinelli, G. Scanu & C. Sollazzo, 2006. Elementi di base per la definizione di una tipologia per i fiumi italiani in applicazione della direttiva 2000/60/EC. Notiziario dei Metodi Analitici IRSA-CNR 2006 (1): 2-19. http://www.life-inhabit.it/cnr-irsa-activities/en/download/all-files/doc_download/3-notiziario-irsa-dicembre-2006

MATTM, 2008. Decreto del Ministero dell'Ambiente e della Tutela del Territorio e del Mare 16 giugno 2008, n. 131: Regolamento recante i criteri tecnici per la caratterizzazione dei corpi idrici (tipizzazione, individuazione dei corpi idrici, analisi delle pressioni) per la modifica delle norme tecniche del decreto legislativo 3 aprile 2006, n. 152, recante: «Norme in materia ambientale», predisposto ai sensi dell'articolo 75, comma 4, dello stesso decreto. Gazzetta Ufficiale 187 suppl. ord. n. 189 del 11 agosto 2008. https://www.gazzettaufficiale.it/eli/id/2008/08/11/008G0147/sg
